# A Neural Computation for Visual Acuity in the Presence of Eye Movements 

**DOI:** 10.1371/journal.pbio.0050331

**Published:** 2007-12-27

**Authors:** Xaq Pitkow, Haim Sompolinsky, Markus Meister

**Affiliations:** 1 Program in Biophysics, Harvard University, Cambridge, Massachusetts, United States of America; 2 Racah Institute of Physics and Center for Neural Computation, Hebrew University, Jerusalem, Israel; 3 Center for Brain Science, Harvard University, Cambridge, Massachusetts, United States of America; 4 Department of Molecular and Cellular Biology, Harvard University, Cambridge, Massachusetts, United States of America; Istituto di Neurofisiologia, Italy

## Abstract

Humans can distinguish visual stimuli that differ by features the size of only a few photoreceptors. This is possible despite the incessant image motion due to fixational eye movements, which can be many times larger than the features to be distinguished. To perform well, the brain must identify the retinal firing patterns induced by the stimulus while discounting similar patterns caused by spontaneous retinal activity. This is a challenge since the trajectory of the eye movements, and consequently, the stimulus position, are unknown. We derive a decision rule for using retinal spike trains to discriminate between two stimuli, given that their retinal image moves with an unknown random walk trajectory. This algorithm dynamically estimates the probability of the stimulus at different retinal locations, and uses this to modulate the influence of retinal spikes acquired later. Applied to a simple orientation-discrimination task, the algorithm performance is consistent with human acuity, whereas naive strategies that neglect eye movements perform much worse. We then show how a simple, biologically plausible neural network could implement this algorithm using a local, activity-dependent gain and lateral interactions approximately matched to the statistics of eye movements. Finally, we discuss evidence that such a network could be operating in the primary visual cortex.

## Author Summary

Like a camera, the eye projects an image of the world onto our retina. But unlike a camera, the eye continues to execute small, random movements, even when we fix our gaze. Consequently, the projected image jitters over the retina. In a camera, such jitter leads to a blurred image on the film. Interestingly, our visual acuity is many times sharper than expected from the motion blur. Apparently, the brain uses an active process to track the image through its jittering motion across the retina. Here, we propose an algorithm for how this can be accomplished. The algorithm uses realistic spike responses of optic nerve fibers to reconstruct the visual image, and requires no knowledge of the eye movement trajectory. Its performance can account for human visual acuity. Furthermore, we show that this algorithm could be implemented biologically by the neural circuits of primary visual cortex.

## Abstract 3

Even when we hold our gaze still, small eye movements jitter the visual image of the world across the retina. The authors show how a stable and sharp image might be recovered through neural processing in the visual cortex.

## Introduction

**Figure 1:**
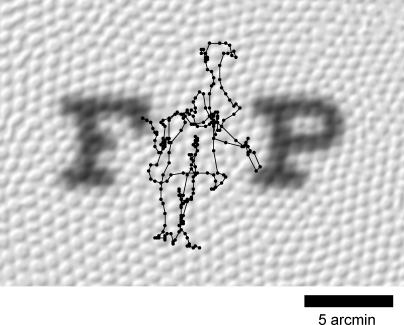
The Neighboring Letters “F” and “P” on the 20/20 Line of the Snellen Eye Chart, Blurred by a Gaussian of Diameter 0.5 arcmin and Projected onto an Image of the Foveal Cone Mosaic (Photoreceptor Image Modified from [92]) The 1-arcmin features that distinguish the letters extend over only a few photoreceptors. Also shown is a sample fixational eye movement trajectory for a standing subject (courtesy of [25]), sampled every 2 ms for a duration of 500 ms and then smoothed with a 4-ms boxcar filter.

People with normal visual acuity are able to resolve visual features that subtend a single arc minute of visual angle. For the letters “F” and “P” on a Snellen eye chart, this corresponds to a difference of just a few photoreceptors (Figure 1). As we try to resolve these tiny features, fixational eye movements jitter them across the retina over distances substantially greater than the features themselves (Figure 1). How can we have such fine acuity when our eyes are moving so much?

If the brain knew the complex eye movement trajectory, then it could realign the retinal responses before processing them further. However, central visual circuits probably do not have access to the eye movement trajectory at a sufficiently fine scale. Fixational eye movements arise from imperfect compensation for head and body movements [1,2] and motor noise [3], so it is unlikely that the visual system has a reliable estimate of the resultant image motion. Although there are both efference copies of eye movement signals and proprioceptive feedback, they have a limited accuracy of several degrees [4,5], which is inadequate for tracking the much smaller movements during fixation. Thus, any estimate the brain makes about fine fixational eye movements is probably driven by visual input alone [6,7].

Unfortunately, visual processing in the retina introduces noise, leaving the brain with uncertainty both about the stimulus shape itself and about the precise trajectory the stimulus traces on the retina. The retina's output neurons—the retinal ganglion cells—are not perfectly reliable in their response to stimulation, and even without stimulation, they fire action potentials at a substantial rate. For brief, small stimuli on a featureless background, the total stimulated retinal response may consist of just a few tens of spikes. The brain must distinguish these spikes from the many hundreds of spontaneous spikes that reflect only noise. The usual remedy would be to accumulate many spikes over time until the signal emerges from the noise; but this is difficult because the fixational eye movements scatter the desired responses across space.

Thus we recognize a challenge for visual acuity in the presence of eye movements: To identify the stimulus, the brain needs to know the precise stimulus trajectory; yet to track the stimulus trajectory, the brain needs to identify which neural spikes are stimulated and which are only noise.

Presented with this challenge, what strategy could the brain use to achieve the visual acuity that humans exhibit? We will show that naive decodings of retinal spike trains that neglect the eye movements perform poorly at discriminating fine visual features. We derive a significantly better strategy that exploits the fact that eye movements are continuous to estimate the stimulus position on the retina and give greater weight to retinal spikes originating near this position. Surprisingly, we found that this strategy is attainable by a simple neural network whose properties are consistent with functional and anatomical features of primary visual cortex.

## Results

### Psychophysics

**Figure 2:**
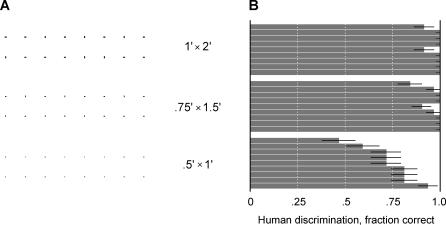
The Discrimination Task (A) Tiny horizontal and vertical stimuli, sized to subtend 0.5 × 1, 0.75 × 1.5, and 1 × 2 arcmin^2^ when viewed at a distance of 88 cm. (B) Performance of nine human participants on this task, measured by the fraction of correct guesses out of 32 trials. Error bars represent the 68% confidence interval.

For concreteness, we choose a simple task to analyze: An observer is asked to discriminate between two tiny oriented bars that span 1 or 2 arcmin of visual angle. In the retina's fovea, this stimulus affects just a few cone photoreceptors, each collecting light from a region about 0.5 arcmin in diameter. Each cone drives approximately one On-type and one Off-type ganglion cell, and conversely, each ganglion cell receives its input from just one cone [8]. This means that at any given instant, the brain receives information about the stimulus from spiking in a small cluster of retinal ganglion cells, but the identity of those cells changes continually as the stimulus jitters across the retina. We tested human subjects on this discrimination task and found that despite these challenges, many human subjects can actually perform well above chance (Figure 2, see also [9,10]).

It is plausible that the finest human acuity might be limited primarily by the information available in the retina rather than by later constraints or losses. For example, our ability to detect dim lights in absolute darkness is ultimately limited by photon shot noise at the rod photoreceptor. In bright light—the condition considered here—noise introduced by retinal processing greatly exceeds photon shot noise [11–13]. Correspondingly, human thresholds on fine acuity tasks are worse by a factor of ten than expected from ideal processing of photon counts [9,10]. Instead, human performance on simple visual tasks is more compatible with the limitations from noisy retinal ganglion cell spikes [14,15]. If acuity is in fact limited by the retinal spike trains, then the brain must make efficient use of these spikes to extract the relevant information.

### Markov Decoder Model

We now present a strategy for accumulating information about position and orientation of the small stimulus bar on the retina. This strategy decodes the observed spike trains from retinal ganglion cells using prior knowledge about the statistics of those spikes and the statistics of eye movements. The output of the decoder is a moment-to-moment estimate of the bar's orientation.

**Figure 3:**
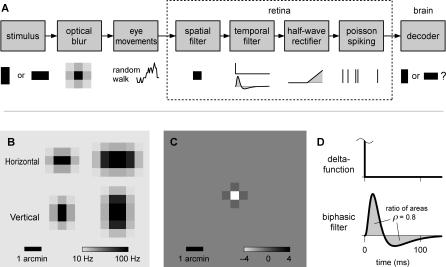
Models of Spike Generation and Decoding (A) A block diagram of the features in the model visual system; see text for details. (B) Firing-rate profiles *r_S_*(**y**) induced by horizontal and vertical stimuli on the model foveal lattice. Left: 0.5 × 1 arcmin^2^. Right: 1 × 2 arcmin^2^. (C) A graphical representation of the discrete second-derivative operator used to calculate diffusion rates. (D) The temporal filters that model retinal ganglion cells use to convert the time-varying light intensity into the instantaneous firing rate.

The decoder assumes a model of retinal ganglion cell spike generation, shown in Figure 3A, which includes random eye movements, optical blur, spatial receptive fields, temporal filtering, rectification, and probabilistic spiking. Each stimulus is a small, dark, oriented rectangle that jitters across the retina. The eye's optics introduce a spatial blur, implemented by a Gaussian filter with a 0.5 arcmin diameter. We assume this image is sensed by photoreceptors arranged on a square lattice, each activating one Off-type ganglion cell. We neglect the On-type cells because they will generate only a weak response to the small, dark stimulus (see Discussion). For the same reason, we neglect the broad, but shallow, surrounds of Off-cells, which are usually approximately 50 times weaker than the receptive field center [16]. Furthermore, we first assume for simplicity that ganglion cells report on the instantaneous light intensity in their receptive field center; later, we will consider implications of including a temporal filter like that in Figure 3D. Under these assumptions, when a stimulus with orientation *S* is at position **x**, a model retinal ganglion cell at position **y** fires action potentials with Poisson statistics at the instantaneous time-dependent rate *r_S_*(**y** − **x**) depicted in Figure 3B, ranging from a peak value *r*_max_ at positions near the stimulus to the background firing rate *r*_0_ at large distances. In bright conditions, retinal ganglion cells respond to a contrast of 100% (black on white) with a spike rate of *r*_max_ ∼ 100 Hz [17]. Far from the stimulus, we assume neurons fire spontaneously with rates on the order of *r*_0_ ∼ 10 Hz [18,19].

In weighting the retinal responses properly, the decoder takes into account the statistics of the trajectories that are traced by the fixed stimulus on the moving retina. Fixational eye movements are classified into three types of motion: microsaccades, drift, and tremor [20]. Microsaccades are not thought to play a role in fine visual tasks [21–23], though they may contribute to peripheral vision [24]. Tremor has very low amplitude, less than a photoreceptor diameter. We therefore concentrate on the drift component, which has the properties of a random walk [3], with modest deviations on short and long timescales [25]. For simplicity, we assume that the fixational eye movements are described by a spatially discrete random walk across the photoreceptor lattice with an effective diffusion constant of *D* ∼ 100 arcmin^2^/s (see Materials and Methods).

For a random walk trajectory, the probability of the current position depends only on its most recent previous position. This attribute, in combination with the assumption that retinal responses are memoryless, allows us to write a differential equation for the probability distribution 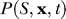 of the stimulus orientation *S* and current location **x**, given all the spikes observed before time *t*:

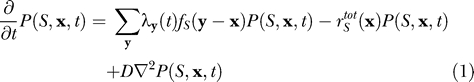
(see Protocol S1 for a derivation). In this equation, 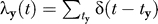 stands for the observed spike train of the retinal neuron **y** at time *t*; 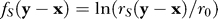 reflects the expected firing-rate profile generated by the stimulus; 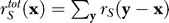 denotes the total expected firing rate of the retinal ganglion cell array; and 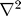 represents a discrete version of a second-order spatial derivative operator. On a square lattice, ∇^2^ 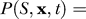 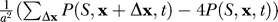 , where **x** + Δ**x** ranges over the four nearest neighbors of **x** on the lattice (Figure 3C), and *a* is the distance between lattice points.

Equation 1, also known as a Fokker-Planck equation, describes a reaction-diffusion system [26]. There are three sources of changes in the stimulus posterior probabilities 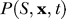 . The first term,

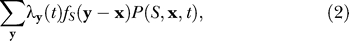
implies that each spike of a retinal neuron **y** results in a multiplicative update of the stimulus posterior probabilities 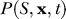 by a factor 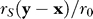 (as shown in Materials and Methods), thus increasing the likelihoods of stimulus positions **x** near the firing retinal neuron, where 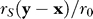 is large. The second term,

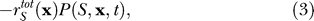
represents the “negative” evidence accumulating during quiescent periods. In between retinal spikes, 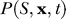 decays exponentially with a decay rate that equals the total expected firing rate of the retinal array with the stimulus *S* at position** x**. In the present case, in which the total activation of the retina is the same regardless of the orientation and position of the stimulus, we ignore this term since it does not affect the relative values of the posterior distribution for different orientations *S* or positions **x**. These first two terms represent the local “reaction” terms. The last term,

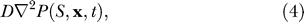
is the “diffusion” term; it describes the lateral spread of the posterior probability across the retina during the time between retinal spikes. This spread accounts for the expected stimulus movements due to the fixational eye movements. The rate of spread is given by *D*, the diffusion constant of the fixational eye movements. The initial condition for solving Equation 1 is specified by 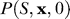 , which is the initial probability distribution of the stimulus orientation and position prior to observing any spikes. We will assume that it is uniform over the entire range of positions and orientations. Finally, we note that Equation 1 technically yields the posterior probability only up to an overall normalization factor (see Protocol S1 for a strictly normalized version). This is unimportant for discrimination, since only the relative values of *P* for different orientations matter. However, in numerical work, one must supplement Equation 1 by a divisive normalization, periodically dividing all components of *P* by the sum 
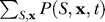
 over space and orientation (see Materials and Methods).

This decoder of retinal spike trains can be applied to a variety of tasks. For instance, in a localization task with a stimulus of known orientation, *S*, the estimate of the stimulus position **x** is given by 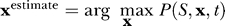 . In a discrimination task in which only the orientation needs to be determined, a sum over the irrelevant position variable yields 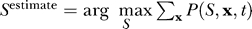 .

The first-order differential equation (Equation 1) implies that the posterior probability can be updated in a way that depends only on the current posterior probability and the current evidence from spikes. This is possible because the assumed process of generating spikes depends only on the current stimulus location. This is an example of what is known as a Markov process, more specifically, a hidden Markov process because the location variable is not observed directly. We will call this decoder of the spike trains the “Markov decoder.” It will yield optimal decisions if the Markov assumptions accurately describe the spike generation process.

### Visualizing the Markov Decoder Algorithm

**Figure 4:**
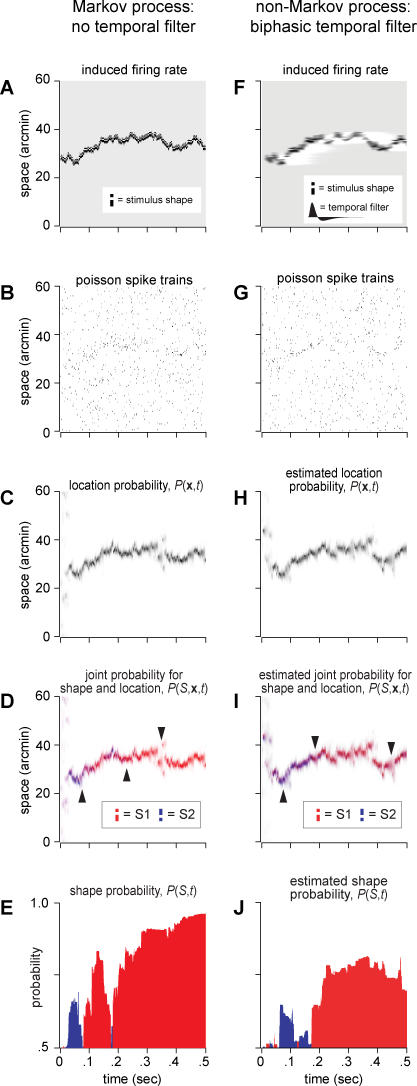
Simulations of the Markov Decoder (Equation 1) for a Small Stimulus Moving on a One-Dimensional Model Retina (A–E) Spike generation by a Markov process. (F–J) Spike generation by a non-Markov process that includes the biphasic temporal filter from Figure 3D. (A and F) Firing rate induced by a stimulus moving on the retina with a random walk diffusion constant of 100 arcmin^2^/s. The stimulus shape activates three neurons in the pattern shown in the inset. The background rate is 10 Hz, and the peak stimulated rate is 100 Hz. (B and G) Poisson retinal spike trains drawn from this instantaneous firing rate. Each row corresponds to a neuron, spaced every 0.5 arcmin. (C and H) Evolution of the location probability 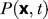 for a known stimulus shape *S* (inset in [A]), but an unknown location **x**, derived from the spike trains shown in the previous panel. (D and I) Decoder behavior when the stimulus can instead take one of two possible shapes, but the true shape is unknown. The two stimuli each activate three retinal neurons, in mirror-image patterns (inset). The spike trains now induce two spatial distributions of the posterior probability 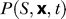 , plotted in shades of red and blue. (E and J) Shape probability 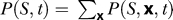 , colored red for the correct stimulus identity and blue for the incorrect one. In these trials, we see that once the decoder coalesces around the stimulus location, it first attributes a greater probability to the wrong stimulus (leftmost arrow in [D] and [I]) before accumulating enough evidence for the correct stimulus (middle arrow). The decoder can lose track of the stimulus briefly (e.g., at rightmost arrow) but continues to favor the correct stimulus until the end of the trial. Note that (E) reflects the true posterior probabilities, whereas in (J), the Markov decoder can only estimate them because the spike generation process includes temporal filtering that the decoder neglects.

We illustrate the performance of the decoder in Figure 4A–4E, using spike trains from a one-dimensional model retina. In the first task (Figure 4A–4C), the stimulus shape is known, so the only uncertainty is its location. The stimulus follows a random walk trajectory, generating the instantaneous firing-rate pattern (Figure 4A), and eliciting extra spikes for neurons along its path while other neurons produce spontaneous spikes at a lower rate (Figure 4B). The Markov decoder collects all the retinal spikes and solves Equation 1 to estimate the posterior probability distribution over positions (see Materials and Methods for numerical details). The result is displayed in Figure 4C.

In the particular trial depicted, the task of localizing the stimulus appears quite difficult, even with only one spatial dimension: In any given time slice, the evidence provided by retinal spikes is rather weak. Nonetheless, the accumulated evidence over time provides a good estimate of the stimulus trajectory. As evidence from the spiking neurons accumulates, the decoder locks onto and tracks the true stimulus location.

In a second task, the decoder must discriminate between two possible stimulus shapes moving on a one-dimensional retina (Figure 4D and 4E). Because one dimension does not allow for horizontal and vertical bars, we take the shape variable *S* to refer to two stimuli related by reflection (Figure 4D, inset). Again, these probabilities evolve according to the reaction-diffusion dynamics of Equation 1, where incoming spikes lead the probability distributions to track the stimulus, but now there is a competition for probability between two stimulus shapes. The Markov decoder may make errors in position, stimulus identity, or both, depending on the particular spike trains it observed, but on average, it discriminates between the two stimulus shapes with an accuracy well above chance.

### Non-Markovian Spike Generation with Temporal Filtering

So far, we have assumed that the retinal ganglion cells report on the instantaneous light intensity, but this is not realistic. Primate photoreceptors react slowly, with integration times on the order of 25 ms [27], yet the eye movements' diffusion constant of 100 arcmin^2^/s implies that the stimulus typically moves one photoreceptor diameter in under a millisecond. Therefore, the firing of retinal ganglion cells cannot track the light intensity as it fluctuates on this rapid timescale. More realistically, the ganglion cells respond to the light intensity in their receptive field averaged by a biphasic temporal filter like that shown in Figure 3D [28].

This temporal filtering has an important implication: Since the spiking probability depends on an extended history of stimulus positions, the spikes cannot be interpreted optimally by the Markov decoder. One can generalize Equation 1 to derive the optimal decoder in this situation. The posterior probability distribution now extends over all possible random walk trajectories within the temporal range of the filter. There are approximately 10^8^ such trajectories leading up to each stimulus location, and propagating their probability distribution is numerically unwieldy. It also seems improbable that the brain takes such an approach. These arguments apply strictly to the optimal decoder, but there may exist useful and efficient nonoptimal decoders. In fact, we found that the simple Markov decoder still performs well at the discrimination task, despite the mismatch between the encoding process and the decoder's assumptions.

To explore this, we generated retinal ganglion cell spikes (Figure 4F and 4G) with a model that includes a biphasic temporal filter (Figure 3D). The filtering adds a motion smear to the stimulus, which renders the output spike trains more ambiguous. Despite its ignorance of the temporal filtering, the decoder can still track the stimulus location, with a small delay due to the filter (Figure 4H). Furthermore, the decoder successfully accumulates information about the stimulus shape (Figure 4I and 4J).

### Performance of the Markov Decoder

We now evaluate the Markov decoder's performance on the original visual task: to discriminate whether a small jittering bar is oriented horizontally or vertically. Here, we modeled the retina and the decoder using two spatial dimensions and simulated many trials of the discrimination task. For every trial, we selected a random stimulus orientation and trajectory, filtered the instantaneous light intensity with a biphasic temporal filter, rectified the result to calculate the expected firing rates for all retinal neurons over time, and generated Poisson spike trains with these firing rates (Figure 3). We then applied the decoder algorithm to these spike trains by numerically solving Equation 1 and selecting the orientation estimated to be more probable. Performance was quantified as the fraction of trials in which the decoder guessed correctly.

**Figure 5:**
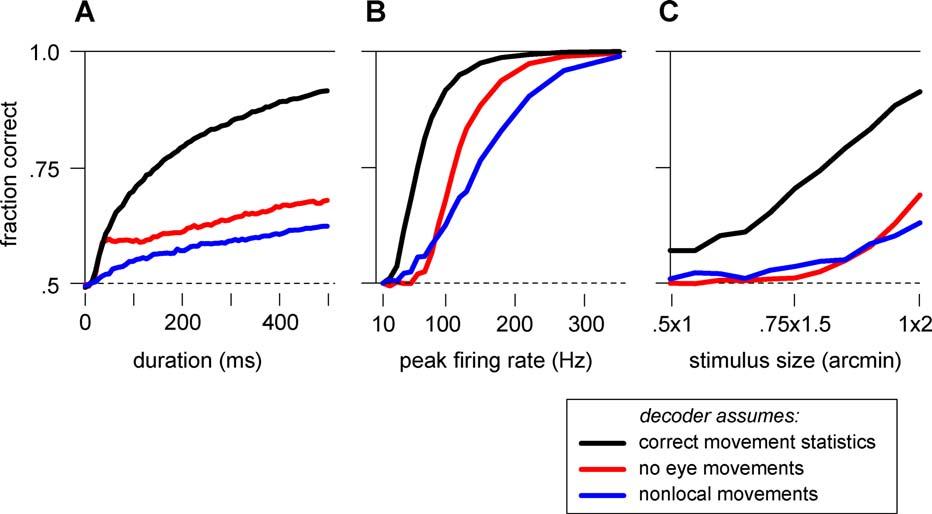
Model Performance on the Horizontal versus Vertical Discrimination Task Shown in Figure 2 Performance is measured by simulating retinal responses, calculating decisions based on those responses, and computing the fraction of correct decisions (see Materials and Methods). When fixational eye movements jitter the stimulus, the Markov decoder is able to perform well on the task by accounting for the eye movement statistics (black curves). Two naive decoders are also applied to this task, one that assumes the stimulus is fixed (red) and one that assumes maximum uncertainty about those movements (blue). Performance is shown as a function of stimulus duration (A), peak stimulated firing rate (B), and stimulus size (C). Where not otherwise specified, the parameters for these simulations are background firing rate of 10 Hz, a peak stimulated rate of 100 Hz, a stimulus of 1 × 2 arcmin^2^, a duration of 500 ms, and a diffusion constant of 100 arcmin^2^/s.

The results of these simulations show that the Markov decoder's performance is generally compatible with human performance. The decoder is able to reliably discriminate horizontal from vertical within a few hundred milliseconds (Figure 5A) using spikes generated at biologically realistic rates around 100 Hz (Figure 5B). Like humans, the Markov decoder finds discrimination very challenging with the smallest stimuli, and fairly routine for the largest (compare Figures 2B and 5C).

### Importance of Accounting for Fixational Eye Movements

The Markov decoder can be used to evaluate the importance of accounting for fixational eye movements in estimating the stimulus shape or orientation. Specifically, we ask the question: how much better does the Markov decoder perform compared to strategies that ignore the eye movement statistics?

Two naive strategies can be proposed: The first assumes that there are no eye movements. This amounts to using a Markov decoder, but setting its presumed diffusion constant to zero. Another strategy recognizes that the eye moves approximately every 0.6 ms (the average time between random walk steps on the square lattice), but is otherwise ignorant of the eye movement statistics; it conservatively assumes that jumps to all stimulus positions are equally likely.

Naturally, the decoder that uses the correct diffusion statistics works best, but simulations reveal that it outperforms the two naive decoders by a large margin (Figure 5). For very brief stimuli of the same duration as the transient retinal response (∼30 ms), the decoder that assumes a fixed stimulus and the decoder that knows the correct movement statistics perform equally well, because temporal filtering does not allow the responses to track the stimulus movements. Yet, under typical viewing conditions, such a duration is too brief for human subjects to discriminate the stimulus shapes. As the decoder integrates information beyond the temporal filter's persistence time, the movements become relevant and the naive algorithm essentially blurs the stimulus even more. The decoder giving equal odds to all locations at all times relies only on the rare coincidences when multiple stimulated neurons spike in tight synchrony. Eventually, this naive decoder can manage to discriminate the stimuli, but it requires a much longer time or many more spikes than the Markov decoder.

### Robustness

**Figure 6:**
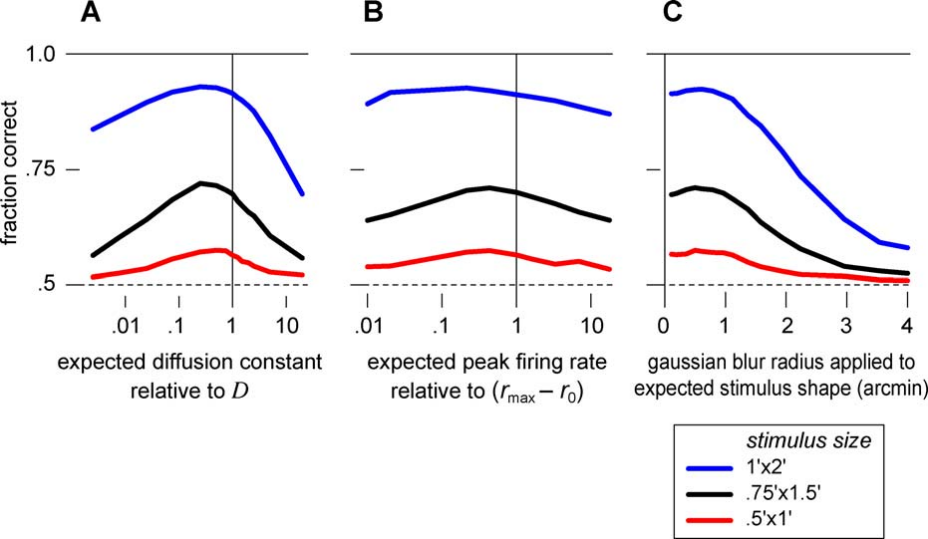
Markov Decoder Robustness to Mismatched Parameters (A) Discrimination performance when the decoder's estimate for the trajectory statistics is wrong: The stimulus is known to perform a random walk on the retina, but the diffusion constant is misestimated. The performance is optimal for estimated values close to the actual diffusion constant and declines gently on either side. (B) Performance as a function of the expected stimulated firing rate, parameterized as 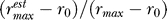 . (C) Performance as a function of the expected stimulus size, obtained by convolving the true stimulus shape with a spatial Gaussian of the specified radius. In each of these plots, parameters are the same as in Figure 5.

How robust is the algorithm to imperfections in implementation? The key parameter that incorporates the statistics of the eye movements is the assumed diffusion constant. As shown above, if the decoder assumes that the eye movements are much faster or much slower than they really are, then the performance degrades substantially. However, between these two extremes, there is a broad range of assumed diffusion constants that causes only a few percent of extra mistakes (Figure 6A). In fact, the decoder benefits slightly from assuming a lower diffusion constant, probably due to the apparent stimulus persistence caused by temporal blurring. This demonstrates that it is essential to account for eye movements, but the algorithm proposed here is robust to misestimates of the movement statistics.

Every time the decoder receives a retinal spike, the estimated stimulus probability rises locally by a factor proportional to the expected stimulated firing rate divided by the background rate (Materials and Methods, Equation 10), which reflects the confidence in the new information brought by a retinal spike. Changing this factor in the Markov decoder would be expected to alter its performance. However, we found that performance is remarkably insensitive to this variable over a wide range of values (Figure 6B).

Finally, we may ask whether the decoder performance is sensitive to the assumed stimulus shapes. Each retinal spike increases the estimated stimulus probability at all those locations where a stimulus could potentially have caused that spike. If the expected stimuli differ from the true stimuli, then this probability increases over the wrong set of locations, leading to suboptimal performance. To explore this, we set the decoder's expected stimulus shape to be larger than the true shape by various amounts (Figure 6C). Enlargement up to about 1 arcmin produced no noticeable change in the decoder's performance, but larger discrepancies of about 2 arcmin led to significant decline. This behavior can be understood as follows: a misestimate of the stimulus size effectively leads to excessive smearing of the positional information. This must be compared to the diffusional smearing that occurs as the stimulus moves in the typical time between informative spikes, which amounts to approximately 1 arcmin. Thus the Markov decoder is hardly affected by misestimates in stimulus shape smaller than this amount.

In summary, the Markov decoder is robust to various parameters that encompass its a priori assumptions about the stimulus. If the decoder allows activity to diffuse at an approximately correct rate, and expects shapes not dramatically larger than the true stimuli, then it can achieve good discrimination performance.

### Network Implementation

Despite the apparent complexity of the differential equation governing the Markov decoder, its dynamics map directly onto a simple neural network with a structure consistent with many known properties of visual cortex. For clarity, we will first introduce a network that estimates the location probabilities for a given stimulus shape, and then show the extension required for shape discrimination.

**Figure 7:**
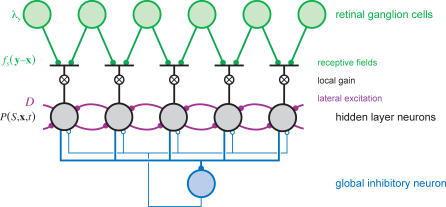
Schematic for a Network Implementation of the Markov Decoder (Equation 1) Spikes from retinal neurons (green, top layer) are collected by neurons in a hidden layer (black, middle layer) with linear receptive fields *f_S_*(**y** − **x**) and a local gain that is set by activity in the recipient neuron. Global divisive inhibition is driven by the total activity of all neurons in the hidden layer through a pooling neuron (blue, bottom neuron).

Figure 7 depicts a network that implements the Markov decoder algorithm for estimating the location of a stimulus with a known orientation *S*. The network has three types of neurons: the retinal neurons, a hidden layer of decoder neurons, and an inhibitory neuron. Each neuron in the hidden layer is associated with a spatial location, **x**, and its activity at time *t* represents the estimated posterior probability (up to a normalization factor) that the stimulus is present at that location, 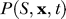 . The feedforward input to each hidden layer neuron **x** consists of spikes from retinal locations **y**, weighted by a spatial receptive field 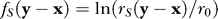 , which ranges from zero far from the stimulus to a peak of 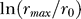 . The weighted retinal input is then multiplied by a variable gain proportional to the activity of the postsynaptic neuron, 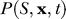 . This gated retinal input implements the contribution of Equation 2 to the update of the estimated posterior probability. The neurons in the network interact through lateral connections mimicking the diffusion operator (Equation 4 and Figure 3C). Recall that the diffusion operator takes the summed probability of the nearest-neighbors of a given location, 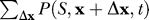 , and subtracts 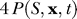 from this in order to conserve probability. In the network, conservation of activity is not required, so the subtraction can be omitted: when the change in *P* is simply proportional to *P*, the solution is an exponential decay that scales *P* uniformly at all locations, leaving the relative values of the activity unaltered. Thus, lateral excitatory connections are sufficient to implement the diffusion term in the network. For the same reason, the network does not need any representation of the local decay term, Equation 3, which also scales all activities equally. Finally, the network includes a global divisive inhibition to maintain network activity at a stable level despite the various excitatory interactions.

**Figure 8:**
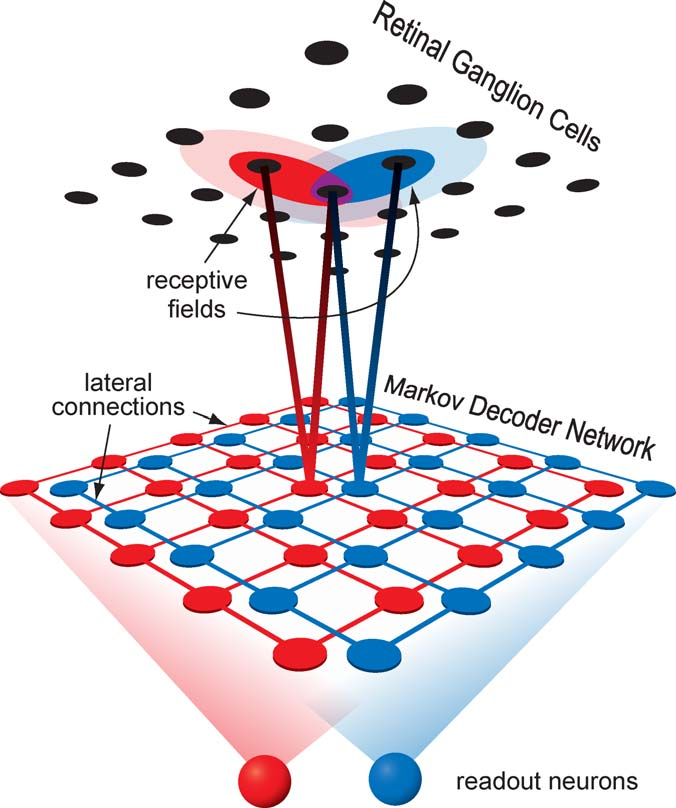
Two Independent but Competing Subnetworks, Each Structured as in Figure 7, Receive Input from the Same Retinal Ganglion Cells, but Use Different Receptive Fields The total activity in each subnetwork is pooled by two readout neurons. The more active readout neuron indicates the network's estimate of the stimulus orientation.

To extend this framework to the discrimination task, we need two copies of the network that differ by their orientation tuning (Figure 8). In the “horizontal” network, representing 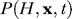 , the neurons are tuned to horizontal stimuli, hence their receptive fields are determined by *r_H_*(**y** − **x**) (Figure 3B); correspondingly, in the “vertical” network, representing 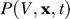 , the receptive fields are related to 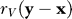 . For the discrimination task, the retinal position is irrelevant; comparing the pooled activity from each subnetwork is sufficient to discriminate between the stimulus orientations. Note that the lateral excitatory connections in this network architecture are orientation specific because fixational eye movements translate the stimulus, but do not appreciably rotate it: orientation, but not position, is preserved. On the other hand, the stabilizing divisive normalization must be global across orientations to ensure a meaningful comparison between the two orientations.

## Discussion

Fixational eye movements pose a major challenge for vision since they scatter weak signals about fine stimulus features across the retina. We addressed this challenge mathematically by deriving an algorithm that guesses the orientation of a stimulus, given spiking responses from a model retina and prior knowledge about its function. It accomplishes this by collecting and sorting the scattered feature information in a systematic way, weighting retinal spikes according to an estimated probability that those spikes reflect stimulus features and not noise.

### Biological Implementation

As described above, the decoder algorithm has a direct mapping onto an abstract neural network, and we will argue that primary visual cortex (V1) has many properties well suited to instantiate this network with real neurons. Specifically, we take the hidden layer neurons in Figure 7 to be cortical cells that receive inputs from the retina via the thalamus.

For good performance, these neurons should integrate retinal spikes using linear, oriented receptive fields of the same shape and size as the visual stimuli (Figure 8). We showed that the decoder's performance was robust to mismatches between the true stimuli and the expected stimuli (Figure 5B and 5C), so these receptive fields need be only approximately tuned to the stimulus size and strength. Linear oriented receptive fields are a well-established characteristic of cortical simple cells [29]. For stimuli subtending only a few human photoreceptors, we require a receptive field of just 1 or 2 arcmin in size. Receptive fields for cortical neurons dedicated to foveal vision are notoriously difficult to measure, notably due to technical problems associated with fixational eye movements. In macaques, receptive fields have been reported as small as 3 arcmin, slightly larger than the macaque's cone resolution of about 1.7 arcmin [30,31]. Therefore, cortical neurons are likely to exist with receptive fields of the appropriate size. Although equivalent measurements are unavailable for human cortex, our finest acuity may well be mediated by cortical neurons driven by an oriented set of just a few cones.

To account for fixational eye movements, the neural network must be organized retinotopically so that local stimulus movements correspond to local interactions in cortex. This is, of course, a known property of V1 [32,33]. Because fixational eye movements are largely independent in each eye [34], the fine retinal positioning of the stimulus is also independent for the two eyes: Proper accounting for stimulus movement, therefore, requires that lateral excitation should not cross eyes. Ocular dominance columns [35] are thus seen as a necessary feature if cortex is to accommodate fixational eye movements. Eye movements are best handled before the signals from the two eyes are mixed, favoring a locus in the lateral geniculate nucleus (LGN) or in V1 for the proposed network.

Eye movements are expected to simply translate visual features, but not rotate them, and these expectations should be built into circuitry. Activity in the model decoder network diffuses across space through lateral excitatory connections between nearby neurons, but only those with similar orientation preferences. In the early visual system, the required iso-orientation facilitation has been observed psychophysically [36–38], anatomically [39–42], and physiologically [43–45]. Lateral diffusion of activity has also been directly imaged in visual cortex [46].

As the eye drifts, the retina moves rigidly in world coordinates. But since the size of cortical receptive fields increases with distance from the fovea [30,47,48], fixational eye movements do not move stimuli across many receptive fields in the periphery. Accordingly, there is no need to compensate for fixational eye movements in the periphery. We expect, therefore, to see some aspects of the cortical network that are specialized for foveal vision. Consistent with this, more of striate cortex is dedicated to responses from the fovea than can be explained by the density of retinal ganglion cells [49–51], and lateral suppression and facilitation differ between central and peripheral vision [37].

The Markov decoder requires that the lateral facilitatory interactions induce localized changes in the gain for new input spikes. Such multiplicative gain modulations have indeed been observed in the visual cortex [52,53]. A number of neural mechanisms have been invoked to create neural multipliers [54–60]. One potential mechanism involves the postsynaptic NMDA (n-methyl-d-aspartic acid) receptor, a glutamate-gated ion channel with a voltage sensitivity that causes it to open only when the postsynaptic potential is sufficiently large. In the visual cortex, NMDA activation has been shown to produce a multiplicative effect on input gain [61]. Synapses between cortical layers and within layers have different NMDA and AMPA (alpha-amino-3-hydroxy-5-methyl-4-isoxazole propionic acid) receptor distributions, so that lateral inputs may be simply additive, whereas feedforward input may experience a variable gain [62], as required by the Markov decoder architecture.

With an accelerating nonlinearity and excitatory interactions, this network has a positive feedback loop that would cause the activity to quickly diverge. Normalization will maintain stability, but the normalization must be global and orientation independent so that neural activities can be compared on the same scale. Previously described wide-field divisive normalization [63–66] can serve this purpose, although other global homeostatic mechanisms would function as well.

In our forced-choice task, the accumulated evidence for the horizontal and vertical stimuli must be compared. This can be accomplished downstream by a final winner-take-all computation in which the total activity in each subnetwork is pooled and then compared [67]. This type of computation must take place somewhere in the brain any time a decision must be reached, and various biological implementations have been proposed for this operation [68,69].

Whereas the input to the network consists of discrete spikes, the network units themselves represent the stimulus probability, which is a continuous variable. This variable might be most simply encoded by the collective firing rate of a cluster of neurons [70], especially given that the number of cells representing the visual field expands dramatically from the retina to the visual cortex [71]. Alternatively, the computation might well proceed with discrete spikes: model networks of spiking neurons tend to produce similar behavior as rate models with continuous variables, so long as the spikes are not too strongly correlated [72].

In summary, all the key elements of a Markov decoder for short line segments are present in the neural circuitry of primary visual cortex. One essential feature, namely monocular processing, is no longer available beyond V1. We therefore propose that V1 functions as a dynamic network to accumulate information on fine stimulus features in the face of fixational eye movements.

### Human Performance versus Model Performance

We presented psychophysical results indicating that human subjects could reliably discriminate between horizontal and vertical stimuli measuring 1 × 2 arcmin (100% accuracy; Figure 2), but that the task was barely achievable when the stimulus was half that size (70% accuracy). Using biologically reasonable parameters, a Markov decoder of retinal spike trains attains comparable, but slightly weaker, performance (90% and 60%, respectively; Figure 5). What additional information do humans have that might account for this discrepancy? Here, we consider several aspects of realistic visual processing that were ignored by the Markov decoder.

We treated only Off-type retinal ganglion cells, but there are equally many On-type cells in the fovea, and in principle, they could also contribute to discrimination. An On cell is suppressed when a small, dark stimulus on a light background enters its receptive field, and is then excited when the stimulus exits. These responses are unreliable because the reduction in firing rate from the background of 10 Hz is detectable only after 100 ms of silence, and the excitatory response is slow and weak. We explored this further with explicit simulation of both On and Off cells: The decoder performance improved very little (unpublished data), less than required to fully account for human acuity.

Human fixational eye movements are not exactly random walks. Instead, they exhibit some small persistence of velocity on a timescale of 2 ms and antipersistence on a timescale of 100 ms [25,73]. To explore how these details affect the Markov decoder's discrimination performance, we performed additional simulations. Antipersistence at long times can be explained by occasional microsaccades that periodically deflect the eye toward its starting position. Such occasional jerks of the image hardly affected a Markov decoder ignorant of microsaccades (unpublished data): After each stimulus jump, there was only a slight delay until the tails of the diffusing posterior distribution encountered the elevated spike rate at the new stimulus location. The persistence of eye movements at short times is consistent with velocity correlations lasting just a few milliseconds [73]. For a given diffusion constant, a persistent random walk lingers longer at each retinal location than a pure Markov random walk, leading to slightly stronger responses. Correspondingly, simulations showed that the Markov decoder's performance improves modestly with the introduction of a short persistence time (unpublished data).

As discussed above, the Markov decoder is suboptimal because of the temporal blurring of the stimulus before spike generation. The optimal decoder must keep track of all possible histories affecting the current firing rate, rather than only the last stimulus position, and the computational effort rapidly becomes prohibitive. Strategies have been proposed to simplify the decoding of such processes [74,75], but these require complicated learning algorithms and do not lend themselves to straightforward neural implementation. A simpler strategy for improving the Markov decoder might be to first process the retinal spike trains with a temporal filter designed to “undo” temporal integration in the retina. This could plausibly take place in the thalamus [76].

Finally, the real visual system enjoys two additional benefits that were not available to the Markov decoder. The first is global image motion: Our human observers viewed the tiny bar stimuli on a white sheet posted within a laboratory scene. As the eye moves, this peripheral background image moves coherently upon the retina, providing additional global motion cues that the brain could perhaps incorporate to improve perception. Second, our model for retinal responses used the most-random spike pattern for a given firing rate, namely a Poisson process. By contrast, real retinal ganglion cells fire more precisely [11,12] and could thus be more informative, even for a Markov decoder.

### Are Fixational Eye Movements Helpful or Harmful?

One commonly held view is that fixational eye movements actually improve vision by preventing the decay of retinal responses that occurs under static stimuli [20]. For example, Rucci and Desbordes have demonstrated that for moderately large, noisy stimuli, orientation discrimination is worse when the image is stabilized on the retina, a result they attribute to a loss of the image motion that would otherwise refresh, and possibly structure, neural activity [77]. In contrast, here we have described these eye movements as a hindrance rather than a help. The transient nature of retinal ganglion cell responses does imply that a fixed stimulus will elicit fewer spikes than a moving stimulus, diminishing the signal that the brain receives. But if the eye movements are too large, then the light intensity is spread thinly over many cells, decreasing each individual response while increasing the positional uncertainty and thus the noise [78]. Between the limits of no eye movement and very large eye movements, an optimum exists. This should occur with eye movements that shift the stimulus to a new set of retinal ganglion cells just as the initial response starts to truncate, and no sooner. For a stimulus area *s* and transient response duration τ, this occurs when the diffusion constant is 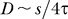 . For tiny stimuli (*s* = 0.5 × 1 arcmin^2^) and biphasic temporal kernels with *τ* = 35 ms, the predicted optimum of *D* ∼ 3 arcmin^2^/s is more than a full order of magnitude smaller than the naturally occurring eye movements of approximately 100 arcmin^2^/s.

**Figure 9:**
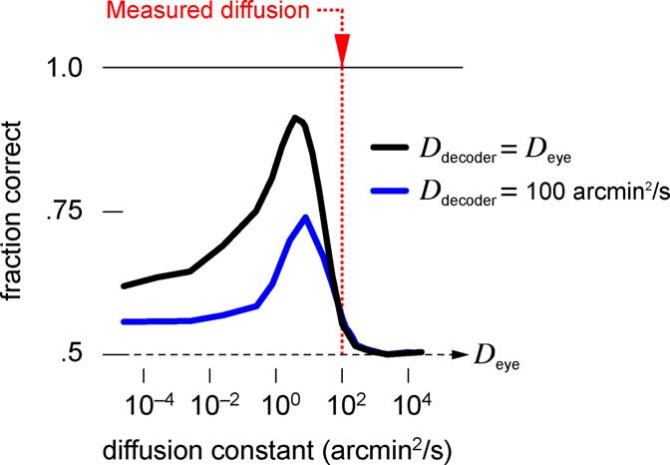
Markov Decoder Discrimination Performance as a Function of Eye Movement Diffusion Constant The decoder's assumed diffusion constant is either held fixed (blue) or covaried with that of the eye (black). The measured diffusion constant for eye movements is marked in red. These simulations used a biphasic filter with perfectly matched positive and negative lobes, which is the filter that most favors large eye movements. The stimulus measured 0.5 × 1 arcmin^2^; otherwise, parameters were as in Figure 5.

To explore this further, we computed the Markov decoder's performance as a function of the eye movement diffusion constant (Figure 9). In one condition, the decoder's assumption about the diffusion constant is held fixed while the eye movement statistics vary; this models a psychophysical experiment in which a viewer's gaze is artificially stabilized. In another condition, the decoder's assumed diffusion constant varies to match the eye movement statistics, approximately optimizing the decoder performance. In both cases, there is an optimum for *D* near the value predicted above, and the model acuity is dramatically worse than this optimum when the natural diffusion statistics are used. Natural eye movements are therefore substantially larger than optimal for this fine acuity task, implying that they do indeed present a problem for fine visual acuity that the brain must solve.

### Predictions

The Markov decoder model yields psychophysical and physiological predictions. We argued that fixational eye movements are unknown to the brain, so using an eye tracker to replace the natural fixational eye movements with exogenous jitter movements, such as eye trajectories recorded from a previous trial, should not affect fine acuity, a prediction supported by recent evidence [79]. We also argued that, for very small stimuli on a featureless background, natural eye movements are larger than optimal: therefore, partially stabilizing the retinal image should improve our finest acuity so long as enough motion remains to avoid prematurely truncating retinal responses (Figure 9). Although discrimination of larger stimuli does benefit from eye movements [79], there are indications that fine acuity is improved by stabilization [80].

There are two major physiological predictions. First, activity in V1 neurons should locally modulate the gain for feedforward input originating from the retina. Without this modulation, the advantage of using prior expectations is lost. Second, if the neural interactions in V1 are to correctly encode the probabilistic expectations given by random walk eye movement statistics, then the interactions should implement a diffusion operator, which entails that the time delay to reach maximal interaction strength should scale as the square of the interaction distance. This should be observable both directly, as lateral excitatory currents, and indirectly, through the time course of the resulting gain modulation.

### The Bayesian Framework

The essential aspect of the Markov decoder we have described is that information of one type attunes the observer to other, related information. In the present context, the decoder expects that responses to oriented line segments are correlated across space and time due to fixational eye movements, and thus these expected responses are enhanced. Other statistical regularities produce expectations as well. For example, strings of line segments often occur together in contours. Correspondingly, collinear iso-orientation facilitation has been hypothesized to subserve contour integration [41,81], and can be viewed as another instance of the principle of enhancing responses to expected signals. More generally, expectations should increase the gain for information that is relevant to the current task, but when that information is irrelevant, then expectations may instead reduce the gain.

The probabilistic processing of information has generated substantial interest as a general framework for neural computation, often designated “Bayesian computation” due to the use of Bayes' rule in calculating probabilities. Human perception has been shown in several conditions to behave according to this rule [82–84]. Experimental evidence also hints that the cortex may be implementing Bayesian inference on a neural level [85]. Modeling studies have suggested how networks of neurons could make these probabilistic inferences [75,86–89]. One study of particular relevance also describes a neural network for approximately Bayesian decoding of arbitrary hidden Markov processes [90].

Although our mathematical formalism is closely related to previous work, we have made several advances in applying the Bayesian paradigm. First, we identified a concrete biological puzzle of considerable practical importance: how can humans see with high acuity when fixational eye movements rapidly jitter the stimulus over a large area? Second, previous Bayesian computations treated neural signals that were poorly constrained by experiment, so the performance of these computations could be characterized only qualitatively. In contrast, retinal signals are well studied, enabling us to make quantitative comparisons between model and human performance. Third, previous studies predominantly described the formal structure of Bayesian computations, whereas we identified a simple and biologically plausible mapping of the probabilistic calculations onto cortical circuitry.

### Outlook

The decoder we have described is optimized for discriminating the orientation of line segments, but human acuity extends to more complex tasks, such as telling “F” from “P.” Within our formalism, optimal discrimination of arbitrary shapes would require receptive fields tuned to those shapes, whereas the early visual system appears to encode oriented edges, with more complex feature selectivity arising only later in higher brain regions. Therefore, this Markov decoder by itself cannot account for discrimination in complex acuity tasks. However, we propose that it functions as a useful preprocessor that reduces the confounding effects of fixational eye movements before passing signals to subsequent cortical regions for high-level processing.

If the stimulus contains several lines of multiple orientations, the decoder's output will have several peaks that correspond to the individual oriented segments. These peaks will track the stimulus pattern as it is scanned over the retina. This output can then be processed by subsequent networks tuned to more complex patterns. Simulations show that such a pattern detector identifies an arrangement of oriented bars better when it is provided with the output of a Markov decoder than with signals from similar decoders that fail to properly account for eye movements (see figure in Protocol S1). Thus, the Markov decoder elaborates the conventional model of V1 as extracting oriented image elements, and improves over this static model through dynamic processing that partially corrects for eye movements.

In real-world acuity tasks, we do not perceive the incessant motion of the image upon our retinas, but rather perceive a stable image in world coordinates. Nonetheless, our internal representation early in the visual pathway stores visual information in a retinal coordinate system [91]. This moving frame of reference must eventually be superseded before our stable perceptions arise and decisions are reached. The network proposed here could be viewed as creating an intermediate coordinate system: the most current information is represented in retinal coordinates, but the nonlinear operations of the network effectively shift the past retinal coordinates into improved alignment. We may view this neural computation as a step towards invariant world coordinates.

## Materials and Methods

### Psychophysics.

Three groups of small horizontal and vertical stimuli like those in Figure 2 were presented at a distance of 4 m and were scaled to subtend the angles 0.5 × 1, 0.75 × 1.5, and 1 × 2 arcmin^2^. Stimuli were printed in black ink on white paper. Ambient lighting generated a luminance of 86 candelas/m^2^ for the white background, and 20-fold dimmer for the black stimuli. Room features provided global motion cues, which we did not seek to eliminate. Nine subjects were asked to discriminate between the stimuli while standing, and were not provided with error feedback. Subjects were free to view the stimuli as long as they liked, typically taking a few seconds per stimulus. Performance was reported as the fraction of correct answers out of 32 attempts for each condition. Error bars were given as 68% confidence interval around the mean, assuming a binomial distribution of correct guesses and a uniform prior over the fraction correct. Other experiments with briefly flashed stimuli showed that reliable discrimination was already achieved within 500 ms (unpublished data).

### Simulations.

We generated model retinal responses for the discrimination task in the following steps: the stimulus orientation *S* was chosen randomly to be either horizontal or vertical, a random walk trajectory was constructed, and the stimulus light intensity profile was moved along this random walk trajectory; the dynamic light intensity at each retinal position was filtered by a temporal kernel, then passed through a threshold rectifier to yield the instantaneous firing rate; this rate drove an inhomogeneous Poisson generator to produce the spike train for the retinal neuron at that location. We passed these spikes to the Markov decoder implementing Equation 1, which returned a guess of the stimulus identity. These steps are depicted in Figure 3A and described in detail below.

In both the simulations of retinal spike trains and in the Markov decoder, we modeled the fovea as a square lattice of cone photoreceptors. In the human retina, cones are spaced every 0.5 arcmin, and the receptive fields of retinal ganglion cells each consist of a single cone. Correspondingly, the model ganglion cells had square receptive fields separated by 0.5 arcmin. For numerical work, we simulated a 16 × 16 arcmin^2^ array with wraparound boundary conditions, which was sufficiently large for the relevant values of the diffusion constant and the diffusion time, yet small enough for fast simulations.

The stimulus itself consisted of a rectangle with size *z* and a 1 × 2 aspect ratio oriented in either the vertical or horizontal direction. Optical blur was produced by convolving the stimulus with a Gaussian modulation transfer function of diameter 2*σ* = 0.5 arcmin [10]. The stimulus at location **x** induced an instantaneous spatial light absorption profile at retinal positions **y** of

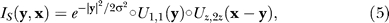
where ○ denotes a convolution operation, and 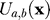 represents a two-dimensional box profile with dimensions *a* and *b*. The resultant stimulus profile is shown in Figure 3B.

We modeled fixational eye movements as a random walk that shifts the stimulus across the retina. The one-sided power spectrum of a one-dimensional random walk is given by 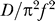 , where *f* is the temporal frequency. Eizenman et al. [3] reported one-sided power spectra with *f* ^−2^ dependence for the horizontal component of fixational eye movements, from which we inferred a two-dimensional diffusion constant of *D* = 100 arcmin^2^/s. Corroborating results come from direct measurements of squared eye displacement as a function of time lag [25]; fitting these data with a straight line of slope 4*D* expected from a random walk yielded diffusion constants of the same magnitude, 100 arcmin^2^/s.

We simulated the trajectory of the stimulus as a random walk on a discrete spatial lattice, but continuous in time. After an infinitesimal time interval *dt*, the probability of stepping to a nearest neighbor location is 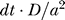 , where *D* is the diffusion constant, and a the distance between lattice points. After many such time steps over a finite interval Δ*t*, the probability that the walker has moved a distance Δ*x* horizontally and Δ*y* vertically can be expressed in series form:

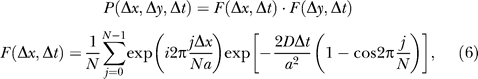
where *N* is the number of points on a side of the square lattice. For speedy simulations, we chose a constant sampling interval Δ*t* = 0.7 ms and drew independent random walk steps from this distribution; finer temporal sampling produced nearly identical results (unpublished data).

The spatial stimulus profile was moved around the model retina according to the random walk. This produced a temporal sequence of light intensities within each retinal ganglion cell's receptive field, which was then convolved with the parameterized biphasic temporal filter (Figure 3D)

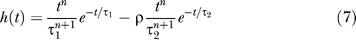
to produce a temporally blurred stimulus (Figure 4F). The parameters were chosen as *τ*_1 _= 5 ms, *τ*_2_ = 15 ms, *n* = 3, and *ρ* = 0.8 for all simulations [28] except Figure 9, for which *ρ* = 1 to maximize the performance improvement attributable to eye movements. Finally, this spatiotemporal profile was offset by the background firing rate *r*_0_, half-wave rectified to prevent negative firing rates, and scaled so that the maximum possible firing rate was given by *r*_max_. The typical firing-rate parameters we used were *r*_0_ = 10 Hz and *r*_max_ = 100 Hz unless otherwise specified.

The Markov decoder operated on one trial of all ganglion cell spike trains to produce a guess for the stimulus identity, according to the differential equation (Equation 1). This equation can be solved iteratively, moving from spike to spike. When neuron **y** produces a spike at time *t* **_y_**, the diffusion term (Equation 4) is negligible compared to the spiking term (Equation 2), so we have only

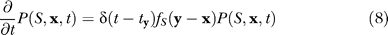



Dividing both sides by 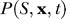 and substituting 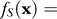 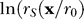 , we see that

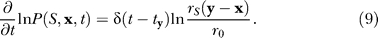



Integrating the delta function over the spike from time 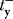 to time 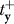 we find that the log-probability jumps at spike times by 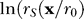 , which means that the probability itself is multiplied:

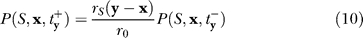



In the absence of spikes, only the terms of Equations 3 and 4 contribute to the differential equation (Equation 1), so the probability distribution 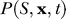 both decays and diffuses laterally across space. Because the two oriented stimuli both produce the same total spike rate from the retinal array regardless of position, the decay term (Equation 3) does not alter the relative probabilities, and we therefore neglect it. The diffusion term (Equation 4) can be implemented most efficiently in the spatial frequency domain 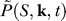 , where the diffusion operator *D* ∇̃^2^ simply multiplies its operand. The solution to

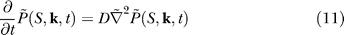
during a spike-free interval [*t*, *t* + Δ*t*] is

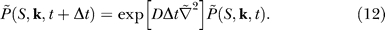



For computational speed, we sampled the decoder's activity every 0.7 ms. Between samples, the probability distribution was multiplied in the Fourier domain according to Equation 12, and at the sample times, the probabilities were multiplied in the spatial domain following Equation 10: once for each spike that occurred since the last sample time. Thus we were able to execute the ideal observer algorithm by multiplication alternately in the spatial domain and the frequency domain. To ensure stability in the absence of the decay term (Equation 3), at every sampling time, we rescaled the posterior probability by its sum, 
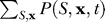
 , recovering a properly normalized probability.

These estimated posterior probabilities can be displayed as a function of space and time, as in Figure 4. Or to reach a decision in the discrimination task, we summed the probabilities over all positions after the specified stimulus duration *T* to obtain the posterior probability for orientation, 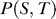 ; the orientation with the greatest probability counted as the decoder's guess. By repeating this process many times (10^4^ iterations) and calculating the fraction of correct trials, we quantified the performance for this ideal strategy for various parameter sets, as plotted in Figures 5, 6, and 9.

## Supporting Information

Protocol S1 The Derivation of the Markov Decoder Equation (1.2 MB PDF)

## Abbreviations

V1: primary visual cortex
