# Supplementary material for: A Neural Computation for Visual Acuity in the Presence of Eye Movements
Source: PLoS Biol. 2007 Dec 27;5(12):e331. doi: 10.1371/journal.pbio.0050331 (PMC2222970; doi:10.1371/journal.pbio.0050331)
Supplement: Protocol S1 — (1.2 MB PDF) [file pbio.0050331.sd001.pdf]

A Neural Computation for Visual Acuity in the Presence of Eye Movements

Xaq Pitkow, Haim Sompolinsky, Markus Meister

– Supporting Material –

Derivation of the Markov decoder equation

In this section we derive the main text's differential equation (1) that describes the Markov decoder for the spike generation process, assuming the retina performs no temporal filtering. We denote by $P\left(S,\mathbf{x}_t \middle| \mathbf{R}_{[0,t]}\right)$ the probability that a stimulus of shape *S* is at location $\mathbf{x}_t$ at time *t* conditioned on $\mathbf{R}_{[0,t]}$, which represents the retinal response for all neurons over the interval $[0,t]$. We first derive an abstract recursive update equation for the probabilities sampled at a finite time interval *dt*, then substitute the particular probabilities given by the spike generation model, and finally move to the continuous time limit $dt \to 0$ that yields Equation (1).

The total response $\mathbf{R}_{[0,t]}$ can be divided into the response $\mathbf{R}_t$ in the current time step and the response history $\mathbf{R}_{[0,t-dt]}$, so that the desired probability is written as

$$P\left(S,\mathbf{x}_t \middle| \mathbf{R}_{[0,t]}\right) = P\left(S,\mathbf{x}_t \middle| \mathbf{R}_t, \mathbf{R}_{[0,t-dt]}\right) \tag{13}$$

We can then use Bayes' rule,

$$P\left(S,\mathbf{x}_t \middle| \mathbf{R}_{[0,t]}\right) = \frac{P\left(\mathbf{R}_t \middle| S,\mathbf{x}_t,\mathbf{R}_{[0,t-dt]}\right) P\left(S,\mathbf{x}_t \middle| \mathbf{R}_{[0,t-dt]}\right)}{P\left(\mathbf{R}_t \middle| \mathbf{R}_{[0,t-dt]}\right)} \tag{14}$$

Since the current responses are assumed to depend only on the present stimulus location, this can be simplified to

$$P\left(S,\mathbf{x}_t \middle| \mathbf{R}_{[0,t]}\right) = \frac{P\left(\mathbf{R}_t \middle| S,\mathbf{x}_t\right) P\left(S,\mathbf{x}_t \middle| \mathbf{R}_{[0,t-dt]}\right)}{Z_t} \tag{15}$$

where $Z_t = P\left(\mathbf{R}_t \middle| \mathbf{R}_{[0,t-dt]}\right)$ ensures the proper normalization of the left hand side. The current stimulus position depends only on the previous position because the underlying process is a random walk, and the stimulus does not change its orientation. Therefore,

$$P\left(S,\mathbf{x}_t \middle| \mathbf{R}_{[0,t-dt]}\right) = \sum_{\mathbf{x}_{t-dt}} P\left(\mathbf{x}_t \middle| \mathbf{x}_{t-dt}\right) P\left(S,\mathbf{x}_{t-dt} \middle| \mathbf{R}_{[0,t-dt]}\right) \tag{16}$$

Combining equations (13)–(16), we obtain the recursive update equation

$$P\left(S,\mathbf{x}_t \middle| \mathbf{R}_{[0,t]}\right) = \frac{1}{Z_t} P\left(\mathbf{R}_t \middle| S,\mathbf{x}_t\right) \sum_{\mathbf{x}_{t-dt}} P\left(\mathbf{x}_t \middle| \mathbf{x}_{t-dt}\right) P\left(S,\mathbf{x}_{t-dt} \middle| \mathbf{R}_{[0,t-dt]}\right) \tag{17}$$

This equation expresses the current posterior stimulus probability as a spatially averaged version of the past stimulus probability, weighted by the current response probabilities and then properly normalized.

Next we substitute the various general probabilities in Equation (17) with the particular probabilities determined by the spike generation model. The factor $P\left(\mathbf{x}_t \middle| \mathbf{x}_{t-dt}\right)$ represents the

probability that the stimulus moves from position $\mathbf{x}_{t-dt}$ to position $\mathbf{x}_t$, which is the probability of a random walk step. This is independent of starting position, so that $P\left(\mathbf{x}_t \middle| \mathbf{x}_{t-dt}\right) = P\left(\mathbf{x}_t - \mathbf{x}_{t-dt}\right)$ and the weighted sum over positions $\sum_{\mathbf{x}_{t-dt}} P\left(\mathbf{x}_t \middle| \mathbf{x}_{t-dt}\right) P\left(S, \mathbf{x}_{t-dt} \middle| \mathbf{R}_{[0,t-dt]}\right)$ becomes a convolution. In an infinitesimal interval *dt*, the probability of moving one step to each of the four nearest neighbor locations is $dt \cdot D/a^2$ and the probability of staying in the same location is $1 - 4dt \cdot D/a^2$, where *D* is the diffusion constant, and *a* is the distance between lattice points. We can use this to express the convolution as

$$\sum_{\mathbf{x}_{t-dt}} P\left(\mathbf{x}_t \middle| \mathbf{x}_{t-dt}\right) P\left(S, \mathbf{x}_{t-dt} \middle| \mathbf{R}_{[0,t-dt]}\right) = \left(1 + D\,dt\nabla^2\right) P\left(S, \mathbf{x}_{t-dt} \middle| \mathbf{R}_{[0,t-dt]}\right) \tag{18}$$

where ∇^2 denotes the discrete second derivative operator and represents a convolution with the kernel

$$k\left(\Delta\mathbf{x}\right) = \frac{1}{a^2} \begin{cases} -4 & \Delta\mathbf{x} = 0 \\ 1 & \left|\Delta\mathbf{x}\right| = a \\ 0 & \text{otherwise} \end{cases} \tag{19}$$

The factor $P\left(\mathbf{R}_t \middle| S, \mathbf{x}_t\right)$ is the likelihood of the stimulus attributes *S* and $\mathbf{x}_t$ based on the response $\mathbf{R}_t$ observed in the current time step. Note that $\mathbf{R}_t$ represents the response from the entire retina. Specifically, we take this to be the vector of spike counts produced by each retinal neuron in the time interval *dt*. With the assumption that all neurons are conditionally independent given the

stimulus orientation and position, the likelihood based on the total retinal response is a product over the likelihoods from each individual spike count $R_{t,\mathbf{y}}$:

$$P\left(\mathbf{R}_t \middle| S, \mathbf{x}_t\right) = \prod_{\mathbf{y}} P\left(R_{t,\mathbf{y}} \middle| S, \mathbf{x}_t\right) \tag{20}$$

A retinal neuron at position $\mathbf{y}$ fires at a rate $r_S(\mathbf{y}-\mathbf{x})$ when stimulus S is centered on position $\mathbf{x}$. Poisson response statistics give the resultant likelihood as

$$P\left(\mathbf{R}_t \middle| S, \mathbf{x}_t\right) = \mathrm{e}^{-r_S^{tot}(\mathbf{x}_t)\,dt} \prod_{\mathbf{y}} \frac{1}{R_{t,\mathbf{y}}!} \left(r_S(\mathbf{y}-\mathbf{x}_t)\,dt\right)^{R_{t,\mathbf{y}}} \tag{21}$$

where $r_S^{tot}(\mathbf{x}) = \sum_{\mathbf{y}} r_S(\mathbf{y}-\mathbf{x})$ is the total retinal mean firing rate induced by a stimulus S at location $\mathbf{x}$.

For the final step in this derivation, we consider the continuous time limit, $dt \to 0$. When the sampling interval dt is small enough, most neurons are silent and at most one retinal neuron at position $\mathbf{y}$ will spike, so that to first order in dt

$$P\left(\mathbf{R}_t \middle| S, \mathbf{x}_t\right) = \begin{cases} r_S\left(\mathbf{y}-\mathbf{x}_t\right)dt & \text{neuron } \mathbf{y} \text{ fires} \\ 1 - r_S^{tot}(\mathbf{x}_t)\,dt & \text{no spikes at time } t \end{cases} \tag{22}$$

Let us first consider the case where no neuron fires at time t, combining Equations (17), (18) and (22) to find that

$$P\left(S, \mathbf{x}_t \middle| \mathbf{R}_{[0,t]}\right) = \frac{1}{Z_t}\left(1 - r_S^{tot}(\mathbf{x}_t)\,dt\right)\left(1 + D\,dt\nabla^2\right) P\left(S, \mathbf{x}_{t-dt} \middle| \mathbf{R}_{[0,t-dt]}\right) \tag{23}$$

The normalization factor Z_t is the sum over all possible states,

$$Z_t = \sum_{S,\mathbf{x}_t} \left(1 - r_S^{tot}(\mathbf{x}_t)\,dt\right)\left(1 + D\,dt\nabla^2\right) P\left(S, \mathbf{x}_{t-dt} \middle| \mathbf{R}_{[0,t-dt]}\right) \tag{24}$$

We can simplify this sum by defining $\overline{r}^{tot} = \sum_{S,\mathbf{x}_t} r_S^{tot}(\mathbf{x}_t) P\left(S, \mathbf{x}_t \middle| \mathbf{R}_{[0,t]}\right)$ and noting that the diffusion kernel totals to zero. Thus to first order in *dt*,

$$\frac{1}{Z_t} = 1 + \overline{r}_S^{tot}(\mathbf{x}_t)\,dt \tag{25}$$

Substituting all relevant terms,

$$P\left(S, \mathbf{x}_t \middle| \mathbf{R}_{[0,t]}\right) = \left(1 + \overline{r}^{tot}\,dt - r_S^{tot}(\mathbf{x}_t)\,dt + dt\,D\nabla^2\right) P\left(S, \mathbf{x}_{t-dt} \middle| \mathbf{R}_{[0,t-dt]}\right) \tag{26}$$

so that finally, writing $P_{t+dt} = P_t + dt\dfrac{dP}{dt}$ we obtain

$$\frac{d}{dt} P\left(S, \mathbf{x}_t \middle| \mathbf{R}_{[0,t]}\right) = \left(\overline{r}^{tot} - r_S^{tot}(\mathbf{x}_t) + D\nabla^2\right) P\left(S, \mathbf{x}_t \middle| \mathbf{R}_{[0,t]}\right) \tag{27}$$

Now let us consider what happens when there is a spike at time *t* by neuron **y**. In this case, Equations (17), (18) and (22) imply that

$$P\left(S, \mathbf{x}_t \middle| \mathbf{R}_{[0,t]}\right) = \frac{1}{Z_t} r_S(\mathbf{y} - \mathbf{x}_t)\,dt\,P\left(S, \mathbf{x}_{t-dt} \middle| \mathbf{R}_{[0,t-dt]}\right) \tag{28}$$

where the diffusion term contributes only to second-order in *dt* and can thus be neglected. The factor Z_t also changes discontinuously, $Z_t = \overline{r}(\mathbf{y})dt$ where we have defined

$\bar{r}(\mathbf{y}) = \sum_{S,\mathbf{x}_t} r_S(\mathbf{y}-\mathbf{x}_t) P\left(S,\mathbf{x}_t \middle| \mathbf{R}_{[0,t-dt]}\right)$. Substituting this normalization into Equation (28) we find that at spike times there is a discontinuous change in *P*,

$$P\left(S,\mathbf{x}_t \middle| \mathbf{R}_{[0,t]}\right) = P\left(S,\mathbf{x}_{t-dt} \middle| \mathbf{R}_{[0,t-dt]}\right) \frac{r_S(\mathbf{y}-\mathbf{x}_t)}{\bar{r}(\mathbf{y})} \tag{29}$$

We can convert Equation (29) to a differential equation by writing it as

$$\frac{d}{dt} \log P\left(S,\mathbf{x}_t \middle| \mathbf{R}_{[0,t]}\right) = \delta\left(t - t_{\mathbf{y}}\right) \log \frac{r_S(\mathbf{y}-\mathbf{x}_t)}{\bar{r}(\mathbf{y})} \tag{30}$$

where $t_{\mathbf{y}}$ is the time of firing of retinal neuron **y** and $\delta\left(t - t_{\mathbf{y}}\right)$ is a Dirac delta-function centered on the spike time.

Combining Equations (27) and (30) we find that, for all times *t*,

$$\frac{d}{dt} P\left(S,\mathbf{x}_t \middle| \mathbf{R}_{[0,t]}\right) = \sum_{\mathbf{y}} \lambda_{\mathbf{y}}(t) \log \frac{r_S(\mathbf{y}-\mathbf{x}_t)}{\bar{r}(\mathbf{y})} P\left(S,\mathbf{x}_t \middle| \mathbf{R}_{[0,t]}\right) + \left(\bar{r}^{tot} - r_S^{tot}(\mathbf{x}_t) + D\nabla^2\right) P\left(S,\mathbf{x}_t \middle| \mathbf{R}_{[0,t]}\right) \tag{31}$$

where $\lambda_{\mathbf{y}}(t) = \sum_{t_{\mathbf{y}}} \delta\left(t - t_{\mathbf{y}}\right)$ is the instantaneous firing rate of neuron **y**.

Using this equation, the probability is always properly normalized. However, we may modify two terms to produce an unnormalized version with slightly nicer properties. In particular, we omit the constant decay term proportional to $\bar{r}^{tot}$, and substitute the background firing rate r_0 in place of $\bar{r}(\mathbf{y})$. This is possible since neither $\bar{r}^{tot}$ nor $\bar{r}(\mathbf{y})$ depend on *S* or $\mathbf{x}_t$, so their

modification does not alter the relative probabilities $P\left(S,\mathbf{x}_t \middle| \mathbf{R}_{[0,t]}\right)$. Defining a weighting for new input spikes,

$$f_S(\mathbf{y}-\mathbf{x}) = \log\frac{r_S(\mathbf{y}-\mathbf{x})}{r_0} \tag{32}$$

which is both invariant over time and spatially localized, we obtain the Markov decoder equation presented in the main text as Equation (1),

$$\frac{d}{dt}P\left(S,\mathbf{x}_t \middle| \mathbf{R}_{[0,t]}\right) = \sum_{\mathbf{y}}\lambda_{\mathbf{y}}(t) f_S(\mathbf{y}-\mathbf{x}_t) P\left(S,\mathbf{x}_t \middle| \mathbf{R}_{[0,t]}\right) - r_S^{tot}(\mathbf{x}_t) P\left(S,\mathbf{x}_t \middle| \mathbf{R}_{[0,t]}\right) + D\nabla^2 P\left(S,\mathbf{x}_t \middle| \mathbf{R}_{[0,t]}\right). \tag{33}$$

Figure S1.

The Markov Decoder Is a Useful Preprocessing Step for Identifying More Complex Shapes Than Single Oriented Bars

Here, we demonstrate this with an example task: discrimination of the two “letters” shown in (A). Each letter is a different configuration of two bars. In a first stage, the stimulus is processed by the coupled network of bar detectors discussed in the text, which produces an array of output signals $P(S,\mathbf{x},t)$. The second stage is a simple translation-invariant pattern detector. It matches the output pattern of the first stage to templates for the two letters, and chooses the letter with the better match. Specifically, we compute

$$Q(L,T)=\prod_{t=1}^{T}\sum_{\mathbf{x}'}\exp\left(\beta\sum_{\mathbf{x},S}f_L\left(S,\mathbf{x}-\mathbf{x}'\right)P\left(S,\mathbf{x},t\right)\right).$$

Here, $P(S,\mathbf{x},t)$ is the output of the Markov decoder; $f_L\left(S,\mathbf{x}-\mathbf{x}'\right)$ is the template of bars with orientations *S* at positions **x** if the letter *L* is located at position $\mathbf{x}'$, as illustrated in (B); β is a weighting factor that determines how accurately the template needs to be matched. After performing the template match over *T* time frames, we compare $Q(L_1,T)$ with $Q(L_2,T)$ and choose the letter with the larger value. The performance of this pattern detector depends strongly on how the retinal inputs are processed by the first stage (C). Here, we vary the effective diffusion constant *D* of the Markov decoder, as in Figure 5: Performance is much better when *D* is adjusted to the eye movement statistics than when *D* is zero (red) or very large (blue). For this plot, the simulation parameters are: background firing rate of 10 Hz, peak firing rate of 150 Hz, stimulus shapes with two 2×0.5 arcmin bars, templates that are zero everywhere except at two

points taking value 1, and weighting factor β = 6. Remaining parameters are the same as in Figure 5.

Figure S1

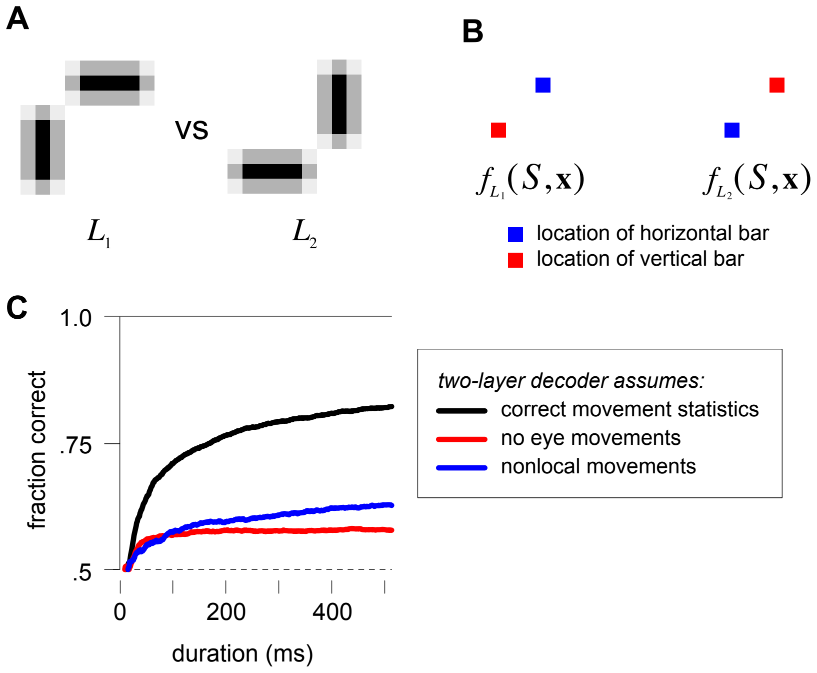
A
vs
L_1
L_2
B
$f_{L_1}(S,\mathbf{x})$
$f_{L_2}(S,\mathbf{x})$
location of horizontal bar
location of vertical bar
C
1.0
.75
.5
fraction correct
0
200
400
duration (ms)
two-layer decoder assumes:
correct movement statistics
no eye movements
nonlocal movements
